# The Sleep Regularity Index: A New Way to Evaluate Shiftwork Schedules

**DOI:** 10.1111/jsr.70133

**Published:** 2025-07-01

**Authors:** Jacob R. Guzzetti, Panagiotis Matsangas, Siobhan Banks, Nita L. Shattuck

**Affiliations:** ^1^ Behaviour‐Brain‐Body Research Centre University of South Australia Adelaide South Australia Australia; ^2^ Crew Endurance Team Naval Postgraduate School Monterey California USA

**Keywords:** maritime defence, sleep restriction, sleep timing, sleep variability, watchkeeping

## Abstract

Sailors adhere to a variety of demanding shiftwork schedules (known as ‘watchbills’) which pose a challenge for sleep and wellbeing at sea. Previous research aimed at identifying viable watchbills based on how they protect sailors' sleep has largely relied on sleep duration. Findings have highlighted insufficient sleep during watchstanding but have mixed results when comparing across watchbills. Sleep regularity is another important dimension of sleep that has been rarely assessed during watchstanding. This study aimed to investigate sleep regularity assessment as a tool for evaluating watchbills, using a recently introduced metric—the Sleep Regularity Index (SRI). Two hundred eighteen sailors from different watchbills were assessed for approximately 1 week of an underway period. The median SRI score was significantly lower for the 5‐h on/15‐h off watchbill compared to all other watchbills, despite no statistically significant differences in daily sleep duration. The median SRI score was similar between fixed watchbills. The large measurable differences in SRI scores between watchbills, particularly when sleep duration was similar, demonstrated the value of sleep regularity assessment as a tool for evaluating watchbills.

## 1 Introduction

Shiftwork is essential to operation in a variety of labour sectors, including healthcare, transportation, and defence. Accordingly, shiftworkers comprise approximately 20% of the industrialised global workforce (Bonnefond et al. 2004). Maritime defence watchstanders are shiftworkers that are exposed to extreme shiftwork schedules, which in the US Navy are called ‘watchbills’. Watchstanders have a particularly demanding life at sea, as the sailors tasked with upholding constant vigilance. Watchbills may be written for the timing of watches to be arranged in a variety of ways, depending on the operational circumstances and resources available on the ship. In efforts to improve sailor wellbeing and minimise the risk of operational errors at sea, studies in the field have investigated the effects of different watchbills on sleep (Kongsvik et al. 2011; Young 2013; Shattuck and Matsangas 2014; Yokeley 2012; Chabal et al. 2024; Shattuck et al. 2014; Shattuck, Matsangas, and Brown 2015; Shattuck, Matsangas, and Powley 2015; Van Puyvelde et al. 2022; Marando et al. 2023).

These studies have largely focused on sleep duration as a primary objective sleep outcome measure. In principle, more watch sections confer less daily time on ‘watch’ and thereby more time available for sleep. However, as the propensity for sleep is largely regulated by the circadian system, the suitability of a given off‐watch period for sleep may vary based on the time of day (Wright Jr et al. 2001). Fixed 12‐ or 24‐h watchbills, unlike rotating non‐24 h watchbills, are compatible with the circadian rhythm, which largely regulates sleep (Wright Jr et al. 2001). However, findings have generated an unclear relationship between watchbill features (e.g., number of sections, fixed or rotating structure) and sleep duration. For instance, the average daily sleep duration for sailors on the fixed two‐section 6‐h on/6‐h off watchbill (or 6/6) has commonly been reported to reach ~5.5 h, which is comparable to that reached on the rotating four‐section 5/15 (Kongsvik et al. 2011; Young 2013; Shattuck and Matsangas 2014; Yokeley 2012; Van Puyvelde et al. 2022; Marando et al. 2023). Moreover, Chabal et al. (2024) observed an average daily sleep duration of 6.62 h for submarine watchstanders at sea on the fixed three‐section 8/16, which was reportedly comparable to the rotating three‐section 6/12. In another at‐sea trial, Shattuck et al. (2014), Shattuck, Matsangas, and Brown (2015), Shattuck, Matsangas, and Powley (2015) and Shattuck and Matsangas (2016) reported the average daily sleep duration for sailors on the rotating three‐section 5/10 to be similar to the fixed four‐section 3/9, but ~1.25 h longer than a modified rotating four‐section 6/18. While the reported effects of different watchbills on sleep duration are inconsistent, in aggregate, these studies have highlighted the ubiquity of obtaining less sleep than is recommended (< 7 h) in watchstanders at sea (Watson et al. 2015). Although, beyond age group‐specific recommendations, interindividual differences in sleep need have been documented (Dashti et al. 2019; Van Dongen et al. 2005). Beyond exposing the margin of sleep deficit that results from standing various watchbills, the assessment of sleep duration may be inadequate for distinguishing the effects of different watchbills on sleep. At any rate, this sleep deficit is likely to result in cumulative performance degradation while underway, placing watchstanders at risk of operational errors (Belenky et al. 2003). Thus, there is a critical need to examine other meaningful sleep outcomes that can inform optimal watchbill selection, such that the consequences of insufficient sleep are not exacerbated. The coupling of sleep duration assessment with an additional objective sleep metric which can generate implications for how various watchbills support sailor performance and wellbeing may be useful.

Sleep regularity is a dimension of sleep that is important for performance and wellbeing; however, it has been neglected in the evaluation of the effects of different watchbills on sleep (Sletten et al. 2023). Circadian disruption, which can result from irregular sleep timing, has long been reported during watchstanding, particularly on rotating watchbills (Naitoh et al. 1983; Plett et al. 1988; Schaefer et al. 1979). As sleep regularity reflects changes in the timing of sleep from 1 day to the next, assessment could offer convenient insight into the magnitude of circadian disruption that may affect watchstanders. While other sleep outcomes are commonly assessed alongside sleep duration in watchbill comparisons (e.g., sleep efficiency, sleep quality), sleep regularity may be distinctly valuable, as the performance consequences of irregular sleep may outweigh those of insufficient sleep (Sletten et al. 2023; Taub and Berger 1973; Johns et al. 1976; Phillips et al. 2017). Watchstanding simulation laboratory studies typically impose strictly scheduled time in bed periods; thus, they may not be conducive to sleep regularity assessment. Historically, assessment of sleep regularity in the field may have been complicated by the frequent observation of sailors splitting their sleep, which convolutes the application of traditional sleep regularity metrics (e.g., sleep midpoint). In one field trial, Beare et al. (1981) applied the coefficient of variation (CV) to assess sleep regularity in watchstanders on the 6/12 and 6/18 watchbills. However, CV is unlikely to be suitable for assessment of sleep regularity in watchstanders, due to the prevalence of splitting sleep into unequal durations (Yokeley 2012; Chabal et al. 2024; Shattuck et al. 2014; Shattuck, Matsangas, and Brown 2015; Shattuck, Matsangas, and Powley 2015; Van Puyvelde et al. 2022; Marando et al. 2023; Beare et al. 1981). CV was computed as the quotient of the standard deviation (SD) of sleep duration divided by the mean (Beare et al. 1981; Baekeland and Hartmann 1971). Thus, the CV for an individual who consistently sleeps from both 23:00 to 05:00 and 13:30 to 15:00 each day would indicate *variable* sleep (CV > zero), despite maintaining a perfectly regularly timed split sleep regime.

The current study therefore aimed to investigate the usefulness of sleep regularity as a sleep characteristic for evaluating watchbills using a recently introduced metric: the Sleep Regularity Index (SRI) (Phillips et al. 2017). A suitable assessment of watchstanders' sleep regularity could be a useful complement to the assessment of sleep duration and other sleep outcomes in the evaluation of watchbills, with no additional strain on the crew. This tool could provide commanding officers with more comprehensive information about how different watchbills may impact their crew's sleep, and ultimately support operational effectiveness at sea.

## 2 Methods

### 2.1 Participants

Active duty crew members from four US Navy surface combatant ships volunteered to participate in the study that occurred during June 2017. All onboard sailors were eligible to participate in the study, with the provision of informed consent. The Institutional Review Board of the Naval Postgraduate School approved the study. Participants performed their typical duties while underway, including training, drills and meeting attendance, among other responsibilities vital to shipboard operations.

### 2.2 Actigraphy

Wrist‐worn actigraphy was used to assess sleep characteristics and patterns, in accordance with recommendations from previous research (Ancoli‐Israel et al. 2015; Morgenthaler et al. 2007). Even though the primary source for the sleep analysis was the actigraphy data, sleep logs assisted in the determination of start and end times of sleep episodes. In particular, we manually identified the start and end times of sleep episodes in the actigraphy data. The actigraphic algorithm automatically assessed sleep variables within each sleep episode.

Two devices were used: the Actiwatch Spectrum (Philips Respironics [PR], Bend, OR) and the Motionlogger Watch (Ambulatory Monitoring Inc. [AMI]). Data were collected in 1‐min epochs for both devices. Action W version 2.7.2155 software using the Cole–Kripke algorithm (with rescoring rules) was used to score AMI data (collected in the zero‐crossing mode). In accordance with default values for this software, the criterion for sleep latency was ≤ 1 min awake in a 20‐min interval, 5 min for sleep and wake episodes. Actiware software version 6.0.0 (PR, Bend, OR) using the medium sensitivity threshold (40 counts/epoch) was used to score PR data. Consistent with software default values, sleep onset and offset were determined by 10 consecutive minutes of immobility. Prior research has demonstrated that these approaches assess sleep time for an ~8 h sleep period at 3‐min accuracy (Meltzer et al. 2012).

### 2.3 Study Design and Procedures

The original observational study employed a prospective, naturalistic design, as data were collected during underway periods in 2017. The current study is a retrospective reanalysis of the data from the original study. The data collection period spanned 9 days on Ship A (with 5–7 days of data per sailor), 10 days on Ships B and C (with 5–9 and 5–8 days of data per sailor, respectively) and 12 days on Ship D (with 5–11 days of data per sailor). Westbound Ships A, C and D each crossed three time zones on Days 5, 6 and 8 of the data collection periods, respectively. Eastbound Ship B crossed one time zone 8 days into the data collection period. For all ships, shipboard time changed with the time zone crossings. No harbours were visited during the data collection periods; thus, sailors' routines were exclusively based on shipboard time. Data collection commenced near the beginning of the underway periods, at which point all sailors had been on their respective routines for at least 3 days.

### 2.4 Analytical Approach

In total, 278 sailors participated in the study. Maintenance shiftworkers (*n* = 15), galley workers (*n* = 15) and sailors who stood multiple watchbills (*n* = 15) or adhered to no set schedule (i.e., ‘work when needed’; *n* = 9) during the study were excluded. Further, a threshold of six contiguous 24 h days of data was established (*n* = 6 excluded), to ensure reliable assessment. Thus, the final analyses included 218 participants.

**FIGURE 1:**
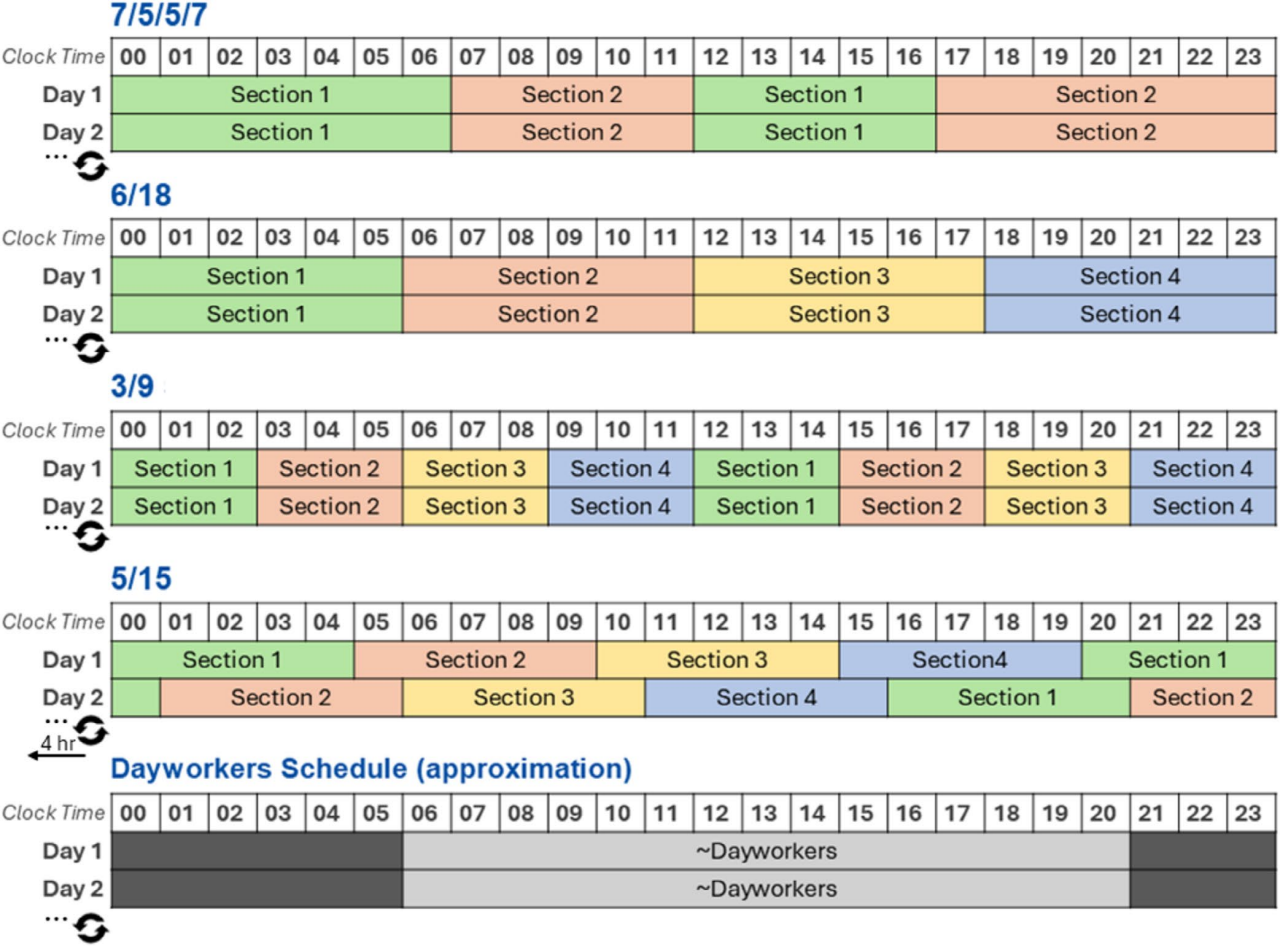
Watchbill rosters.

Participants were primarily watchstanders, whose work involved the execution of specialised responsibilities in a cyclic interval. Until relieved of duty, watchstanders are required to remain at their post. Watchstanders were grouped based on their watchbill, which was either the fixed two‐section 7/5/5/7, fixed three‐section 8/16, fixed three‐section 4/8, fixed four‐section 6/18, fixed four‐section 3/9, rotating four‐section 5/15 or rotating three‐section 5/10 watchbill. A group of non‐watchstanding ‘dayworkers’, whose work occurred during the morning to early evening (consistently allowing nocturnal sleep), were also included for comparison (Figure 1).

Study duration was computed as the difference (i.e., the number of days) between the start and end dates of the data collection period. Analysis of sleep included the following variables: sleep episode duration, wake period duration, daily sleep duration and number of sleep episodes per day. The average sleep episode duration was calculated across all sleep episodes captured during the study. Average wake period duration was calculated across all wake periods, starting at the end of the first captured sleep episode and ending at the beginning of the last captured sleep episode. Sleep episodes and wake periods were aggregated over the data collection period to generate averages for each sailor. For each sailor, daily sleep duration was calculated by first totalling all sleep durations across the study period, respectively. These totals were each subsequently divided by the study duration. The number of sleep episodes per day was calculated as the number of sleep episodes captured during the study divided by study duration. This enabled the calculation of the proportion of sailors that split their sleep (> 1 sleep episode per day). Further analyses of sleep episodes per day only included sailors with split sleep.

The SRI score was used to assess sleep regularity. Unlike conventional sleep regularity metrics (e.g., midpoint of sleep or CV), the SRI does not assume one main sleep episode per day (Phillips et al. 2017; Fischer et al. 2021). The SRI computes the likelihood of binarily being in the same state (wake or sleep) at any two time points 24 h apart (Phillips et al. 2017). In practice, SRI scores can range from −100 to 100, with higher scores indicating more regular sleep, and the metric relies on the capture of all time spent asleep on each 24‐h day used in the calculation. Thus, the first and last calendar days of actigraphy were excluded from the SRI calculation, as the provision and collection of the actigraphy devices occurred on these days, respectively. One SRI score is generated per sailor from their entire data collection period. Despite trans‐meridian travel, all sleep variable calculations are based on the time zone of the port of departure to capture the contiguous 24 h cycles of the study period.

Statistical analysis was conducted using RStudio software version 4.3.1, with the aid of the *dplyr*, *emmeans*, *graphics*, *lubridate, stats* and *tidyr* libraries. Basic descriptive summary statistics were first calculated for all sleep and demographic (sex, age and rank group) variables. Comparisons were then carried out between five groups: dayworkers and watchstanders on the 7/5/5/7, 6/18, 3/9 and 5/15 watchbills. The 4/8, 8/16 and 5/10 watchbills were descriptively summarised and included in correlation analyses but restricted from the group comparisons due to limited group size. Finally, the relationship of SRI score to all other sleep and demographic variables was assessed.

Age and all sleep variables other than daily sleep (*p* = 0.88) were determined to be non‐normally distributed (*p* < 0.05) with the Shapiro–Wilk test. Pairwise Fisher's Exact tests with the Benjamini–Hochberg (BH) post hoc controlling procedure were used to compare the five groups based on sex and the number of sailors with split sleep. Kruskal–Wallis tests and one‐way ANCOVA (ship, sex, rank group and age nested within rank group entered as covariates) were used as appropriate to compare the five groups. Post hoc pairwise comparisons were made using the Dunn test with the BH adjustment or Tukey's honestly significant differences (HSD) test, respectively. Point‐biserial correlations (*r*_pb_) were used to examine the relationship of SRI score to sex and rank group. Spearman correlations (rho) were used to examine the relationship of SRI score to all other sleep variables and age. For all groups except the dayworkers, additional relationships were examined between the SRI score, the number of watch sections and the length of the longest ‘off‐watch’ period. Data are reported as mean (*M*) ± SD or median (MD; interquartile range [IQR]), based on normality. An *α* level of 0.05 was set as the threshold for statistical significance.

## 3 Results

Participants were primarily male (173, 79.5%), enlisted personnel (167, 79.4%), with a median age of 26.5 (IQR = 9). The study sample was demographically representative of the USN active duty service member population (Department of Defense, n.d.). A summary of the demographic characteristics of the participants is shown in Table 1.

**TABLE 1:** Demographic data.

Group	Sections	*n*	Age (years; MD [IQR])	Sex	Rank group
Dayworkers	—	42	29 (14.25)	4 F	6 officers
7/5/5/7 Watchstanders	2	16	23.5 (4.75)	6 F	0 officers
8/16 Watchstanders	3	6	27.5 (4.5)	0 F	0 officers
4/8 Watchstanders	3	5	24 (3)	3 F	0 officers
3/9 Watchstanders	4	70	26.5 (8.75)	22 F	29 officers
6/18 Watchstanders	4	13	27 (8)	1 F	0 officers
5/10 Watchstanders	3	9	24 (6)	1 F	1 officer
5/15 Watchstanders	4	57	26 (8)	8 F	15 officers
Overall	—	218	26.5 (9)	45 (20.6%) F	51 (23.4%) officers

Across all sailors, the median actigraphy‐derived sleep episode duration was 4.46 (IQR = 1.90) h. and the median wake period duration was 11.49 (IQR = 4.56) h. Participants had a daily sleep duration of 6.59 ± 0.99 h (*M ±* SD; ranging from 3.96 to 9.4 h). Eighty‐six percent (*n* = 188) of sailors split their sleep into a median of 1.5 (IQR = 0.57) sleep episodes per day. Participants' SRI scores ranged from 13.14 to 94.91, with a median value of 66.09 (IQR = 28.21). These results are summarised in Table 2.

**TABLE 2:** Overall sample sleep variables.

Variable	Mean	SD	Median	IQR	Minimum	Maximum	Shapiro–Wilk *p*
Sleep episode duration (h)	4.67	1.34	4.46	1.90	1.71	8.50	< 0.001
Wake period duration (h)	11.59	2.98	11.49	4.56	6.19	18.89	0.003
Daily sleep duration (h)	6.59	0.99	6.62	1.30	3.96	9.40	0.882
Sleep episodes per day (#)^a^	1.59	0.36	1.50	0.57	1.10	2.57	< 0.001
SRI (0–100)	61.80	18.15	66.09	28.21	13.14	94.91	< 0.001

^a^ For sailors with split sleep (*n* = 188).

### 3.1 Group Comparisons

Study groups are summarised in Table 3. Ninety‐one percent (*n* = 198) of all sailors were included in the group comparison (Table 1). Of those sailors, 42 (21.2%) were dayworkers, 16 (8%) were on the 7/5/5/7 watchbill, 70 (35.4%) on the 3/9, 13 (6.6%) on the 6/18 and 57 (28.8%) on the 5/15. The groups did not differ based on age or sex (Kruskal–Wallis test, *p* = 0.059; pairwise Fisher's exact test, all *p* > 0.05, respectively).

**TABLE 3:** Sleep variables by group.

Variable	Dayworkers (*n* = 42)	7/5/5/7 (*n* = 16)	8/16 (*n* = 6)^j^	4/8 (*n* = 5)^j^	6/18 (*n* = 13)	3/9 (*n* = 70)	5/10 (*n* = 9)^j^	5/15 (*n* = 57)
Sleep episode duration (h), MD (IQR)^g^	6.17 (1.44)^a^***, ^b^***, ^d^***	3.43 (0.81)^c^***, ^d^***, ^e^***	5.56 (2.10)	3.26 (1.18)	4.91 (1.73)^a^***,^b^**	3.86 (1.39)^c^**, ^d^**^,^ ^e^***	4.02 (0.80)	4.73 (1.27)^a^***, ^b^**, ^e^***
Wake period duration (h), MD (IQR)^g^	13.98 (4.45)^a^***, ^b^***, ^d^**	8.36 (2.51)^b^*, ^c^**, ^d^**, ^e^***	15.11 (4.06)	9.54 (2.93)	11.60 (4.29)^a^**,^b^*	10.36 (4.53)^a^*, ^c^*, ^d^*, ^e^***	12.29 (3.70)	11.74 (1.89)^a^**, ^b^*, ^e^**
Daily sleep duration (h), *M* ± SD^h^	6.96 ± 1.06^d^*	6.81 ± 1.84	6.68 ± 0.35	6.01 ± 0.65	7.01 ± 0.99	6.56 ± 0.94	5.94 ± 1.3	6.91 ± 1.06^e^*
Sailors with split sleep (#) (%)^i^	24 (57.1%)^a^**, ^b^***, ^d^***	16 (100%)^e^**	4 (66.7%)	4 (80%)	9 (69.2%)^b^*	68 (97.1%)^c^*, ^e^***	8 (88.9%)	55 (96.4%)^e^***
Sleep episodes per day (#), MD (IQR)^g^, ^k^	1.29 (0.36)^a^***,^b^**	2.0 (0.54)^d^***, ^e^***	1.29 (0.36)	1.93 (0.31)	1.56 (0.40)	1.71 (0.57)^d^***, ^e^**	1.67 (0.10)	1.33 (0.33)^a^***, ^b^***
SRI (0–100), MD (IQR)^g^	75.19 (15.70)^d^***	65.79 (8.61)^d^***	77.3 (43.26)	72.29 (18.45)	69.84 (19.89)^d^***	71.54 (11.92)^d^***	32.5 (16.63)	43.33 (10.00)^f^***

*Note*: Statistical significance level: *p < 0.05; **p < 0.01; ***p < 0.001.

^a^ Different from ‘7/5/5/7’ group.

^b^ Different from ‘3/9’ group.

^c^ Different from ‘6/18’ group.

^d^ Different from ‘5/15’ group.

^e^ Different from ‘dayworkers’ group.

^f^ Different from all other groups.

^g^ Multiple comparisons with Dunn post hoc test.

^h^ Multiple comparisons with Tukey's HSD post hoc test.

^i^ Pairwise comparisons with Fisher's Exact Test with BH post hoc controlling procedure.

^j^ Group excluded from statistical comparison.

^k^ For sailors with split sleep (*n* = 188).

The 3/9 and 7/5/5/7 groups had a shorter sleep episode duration (3.86 [1.39] and 3.42 [0.81] h, respectively) than the 5/15 (4.73 [1.27] h), 6/18 (4.91 [1.56] h) and dayworkers groups (6.17 [1.44] h, Dunn post hoc test, all *p* < 0.01). The 5/15 group also had a shorter sleep episode duration than the dayworkers group (Dunn post hoc test, *p* < 0.001); only the 6/18 group had a similar sleep episode duration to the dayworkers group (*p* = 0.082). The 7/5/5/7 group had the shortest wake period duration (8.36 [2.52] h), which was significantly shorter than the 3/9 (10.36 [4.53] h), 5/15 (11.74 [1.89] h), 6/18 (11.6 [4.28] h) and dayworkers (13.98 [4.45] h) groups (Dunn post hoc test, all *p* < 0.05). The 3/9 group had a shorter wake period duration than the 5/15, 6/18 and dayworkers groups (Dunn post hoc test, all *p* < 0.05). The 5/15 group also had a shorter wake period duration than the dayworkers group (Dunn post hoc test, *p* = 0.002); only the 6/18 group had a similar wake period duration to the dayworkers group (*p* = 0.246). On average, daily sleep duration for the 5/15 group was ~0.5 h shorter than the dayworkers group (Tukey's HSD post hoc, *p* = 0.031). Proportionately, split sleep was most abundant in the 7/5/5/7 (16 sailors, 100%), 3/9 (68 sailors, 97.1%) and 5/15 groups (55 sailors, 96.4%), whereas significantly fewer sailors split their sleep in the dayworkers group (24 sailors, 57.1%; pairwise Fisher's exact test, all *p* < 0.01). Split sleep was also more abundant in the 3/9 group compared to the 6/18 group (9 sailors, 69.2%; pairwise Fisher's exact test, *p* = 0.034). For sailors with split sleep, there were more sleep episodes per day in the 7/5/5/7 (2.0 [0.54]) and 3/9 (1.71 [0.57]) groups, compared to the 5/15 (1.33 [0.33]) and dayworkers (1.29 [0.36]) groups (Dunn post hoc test, all *p* < 0.01).

The 5/15 group had the lowest SRI of any group (43.33 [10.00] SRI score, Dunn post hoc test, all *p* < 0.001). The SRI score was similar across the 7/5/5/7 (65.79 [8.61]), 3/9 (71.54 [11.92]), 6/18 (69.84 [19.89]) and dayworkers (75.19 [15.7]) groups (Dunn post hoc test, all *p* > 0.05). [Correction added on 12 July 2025, after first online publication: The data “(43.33 [Van Puyvelde et al. 2022] SRI score, Dunn post hoc test, all p < 0.001)” has been corrected to “(43.33 [10.00] SRI score, Dunn post hoc test, all p < 0.001)” in this version.]

### 3.2 SRI Score Relationship to Sleep and Demographic Variables

**FIGURE 2:**
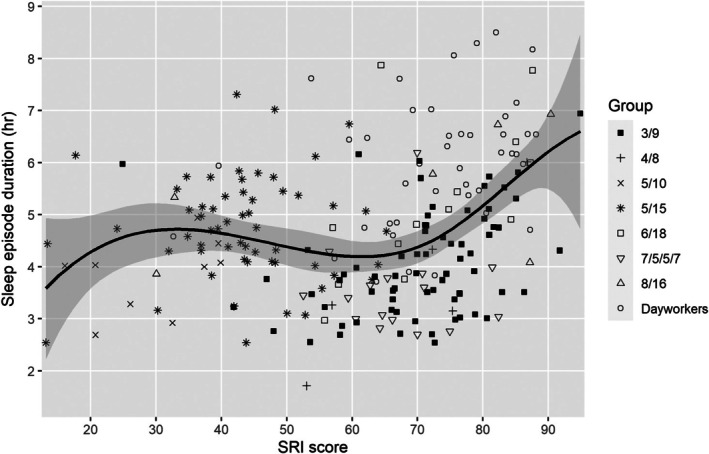
SRI score versus sleep period duration (lambda = 2.0).

**FIGURE 3:**
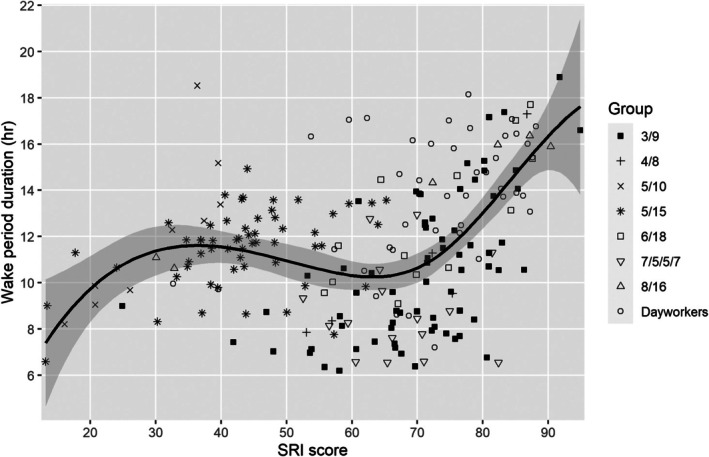
SRI score versus wake period duration (lambda = 2.0).

**FIGURE 4:**
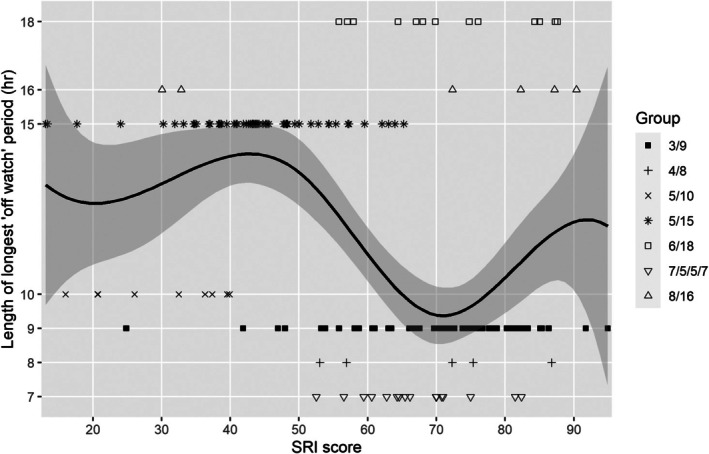
SRI score versus length of longest ‘off watch’ period (lambda = 2.0).

The relationship of SRI score to sleep and demographic variables is summarised in Table 4. Across the study sample, SRI score had no relationship to sex (*r*_pb_ = 0.097, *p* = 0.155), rank group (*r*_pb_ = 0.089, *p* = 0.190) or age (Spearman's rho = 0.101, *p* = 0.139). Sleep episode duration and wake period duration were both correlated with SRI score (Spearman's rho = 0.227 and 0.337, respectively, both *p* < 0.001; Figures 2 and 3). Additional factors were assessed for watchstanders (all sailors other than dayworkers group). For watchstanders, the SRI score was not correlated with the number of watch sections (2–4, Spearman's rho = 0.008, *p* = 0.906) but was negatively correlated with the length of the longest ‘off watch’ period (either 7, 8, 9, 10, 15, 16 or 18 h; Spearman's rho = −0.389, *p* < 0.001; Figure 4).

**TABLE 4:** SRI score correlation to sleep and other demographic variables.

Factor	Coefficient	95% Confidence interval	*p*
Sex	—	—	0.155
Age	—	—	0.139
Rank group	—	—	0.190
Number of watch sections^a^	—	—	0.906
Length of longest ‘off watch’ period^a^	–0.389	–0.496 to –0.27	< 0.001
Sleep episode duration	0.227	0.097 to 0.349	< 0.001
Wake period duration	0.337	0.214 to 0.45	< 0.001
Daily sleep duration	—	—	0.328
Sleep episodes/day^b^	—	—	0.210

^a^ For watchstanding sailors (*n* = 176).

^b^ For sailors with split sleep (*n* = 188).

## 4 Discussion

Based on watchstanding field data, we investigated, for the first time, sleep regularity using the SRI metric as a tool for evaluating watchbills. Previous watchstanding field studies have not assessed sleep regularity or applied metrics that are dependent on a consolidated sleep structure rather than one that allows for naps. We found that sleep regularity differed between watchbills, despite no significant changes in daily sleep duration; in particular, sleep was least regular on the 5/15, a rotating watchbill. Sleep regularity assessment using the SRI was externally validated in this watchstanding population and shown to be a useful supplementary tool for evaluating watchbills, as it expanded meaningful insight into the viability of different watchbills based on how they support sailors' sleep.

We found that sleep regularity was independent of daily sleep duration. This has been previously reported in university students (Phillips et al. 2017), young adult athletes (Alves Facundo et al. 2022; Halson et al. 2022) and a cohort of predominantly working or retired older adults (Lunsford‐Avery et al. 2018). While a study in young non‐nightshift working adults with delayed sleep–wake phase disorder by Murray et al. (2019) and another in athletes by Teece et al. (2024) did find a relationship between sleep regularity and sleep duration, it is likely these populations had less variability in sleep timing at the time of assessment and therefore sleep duration and timing were more consistent. Murray et al. (2019) excluded participants whose work hours fell outside of 0600–2300, and Teece et al. (2024) highlighted that their assessment in professional rugby athletes occurred during a relatively calm period (e.g., no competition or associated travel) In the current study, we focused on assessing sleep regularity during a rigorous period of work in a continuous operational setting, where sleep and work occur at all hours of the day. Phillips et al. (2017) reported a higher frequency of napping in university students with irregular sleep timing. However, we found that sleep regularity had no relationship to the number of sleep episodes per day (accounting for naps), which may reflect the similarly high prevalence of split sleep across all watchbills.

We found that sleep regularity had a positive relationship with both sleep period duration and wake period duration. Together, these findings may suggest that longer consolidated sleep episodes are likely to better support sleep regularity. Compared to short sleep episodes, longer consolidated sleep episodes may better resolve homeostatic sleep pressure, which may otherwise lead to more naps and splitting sleep. In high frequency, this could shorten wake period duration, on average. Interestingly, we found a negative relationship of SRI to the length of the longest ‘off watch’ period for watchstanding sailors. This could suggest that shorter ‘off watch’ periods better support regular sleep timing, in the context of our findings at large. This also might be driven by the large proportion of sailors in this study on the 5/15 watchbill, for whom the ‘off watch’ period was second longest, and sleep was least regular. As watchstanders' sleep regularity had no relationship to the number of watch sections, the total duration of time spent on watch each day may have a negligible impact on sleep regularity.

In terms of other sleep metrics commonly applied in watchstanding field studies, our findings at the population level were consistent with previous assessments of underway sailors, which have highlighted the prevalence of split sleep totalling < 7 total hours daily (Shattuck and Matsangas 2014; Yokeley 2012; Chabal et al. 2024; Shattuck et al. 2014; Shattuck, Matsangas, and Brown 2015; Shattuck, Matsangas, and Powley 2015; Van Puyvelde et al. 2022). Differences in sleep episode and wake period duration between watchbills in the present study were likely dictated by the constraints of watchbill temporal structure. For instance, the shortest sleep episode durations were observed for the 7/5/5/7 and 3/9 watchbills, which offered the shortest ‘off watch’ periods of the watchbills compared (5 or 7 h, and 9 h, respectively). These constraints also likely underscored the observation of the shortest wake period duration for the 7/5/5/7 watchbill. Split sleep prevalence for the 6/18 watchbill (with the longest ‘off watch’ of 18 h) was relatively low among the watchbills and similar to the non‐watchstanding dayworkers. This observation may have been underpinned by features of the 6/18 watchbill which better promote consolidated sleep, including the fixed 24‐h structure with a single watch and a long ‘off watch’ period. Alternatively, split sleep was ubiquitous on the 5/15 (also with a long ‘off watch’ period of 15 h), and similar to that of the 7/5/5/7 and 3/9 (each with shorter ‘off watch’ periods). This observation may reflect an increased need to compensate for sleep loss on a watchbill that is not circadian‐aligned (i.e., based on a 24‐h day) (Kim et al. 2023). However, while an overwhelming majority of sailors split their sleep on the 5/15, 3/9 and 7/5/5/7 watchbills, sailors on the 5/15 appeared to do so less frequently compared to the 3/9 and 7/5/5/7. This may reflect challenges to initiating sleep associated with the circadian misalignment precipitated by the 5/15, though a relatively longer 15 h ‘off watch’ period which could support a long, consolidated sleep episode may have also contributed to this observation (Kim et al. 2023; Baron and Reid 2014).

Sleep duration is typically a key sleep outcome considered in terms of how different watchbills support sleep and ultimately operational effectiveness. In the current study, watchstanders from all watchbills examined were exposed to chronic sleep restriction, consistent with previous watchstanding field studies. Importantly, even though sailors on the 5/15 watchbill were able to obtain a similar daily sleep duration to the other watchbills, their sleep was considerably less regular. For sailors on the 5/15 watchbill, the detriments from sleep loss were likely exacerbated by the consequences of irregular sleep timing, including degraded cognitive inhibition and psychomotor performance, and mood impairment (Gilmore et al. 2024; Taub 1978). The unique non‐24 h rotating structure of the 5/15 watchbill likely contributed to less regular sleep, as the ‘off watch’ time when sleep may occur was shifted backwards (counterclockwise) by 4 h from one day to the next. Thus, the highly irregular sleep timing for the 5/15 may suggest that circadian disruption was worst on this watchbill.

**FIGURE 5:**
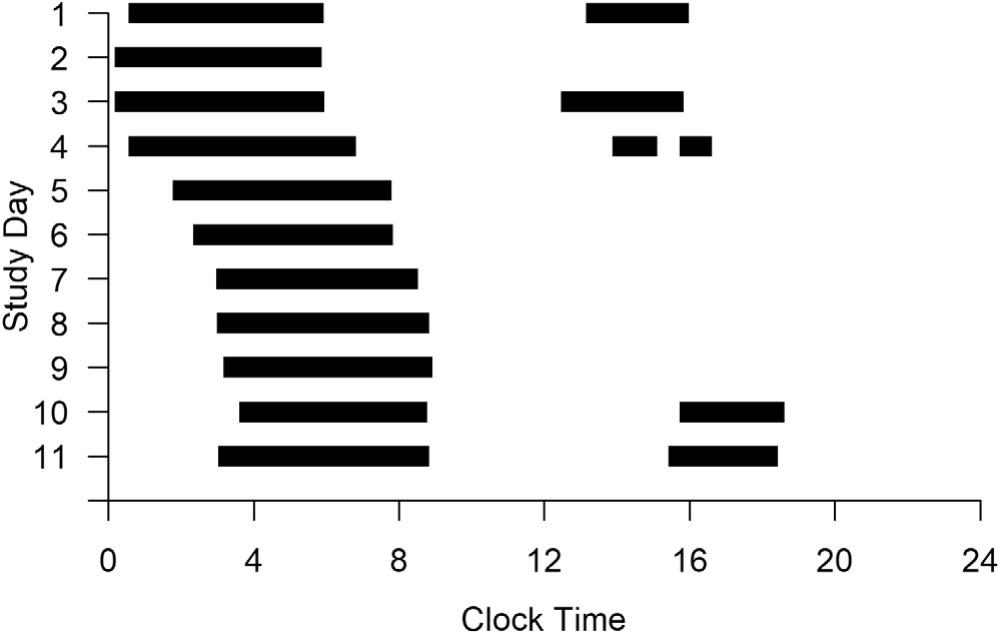
Sleep timing for a sailor on the fixed 7/5/5/7 watchbill. *Note*: The plot is based on time at the port of departure. Black bars indicate sleep timing. Watch duty occurred 07:00–12:00 and 17:00–00:00. Westward time zone crossings occurred on Study Days 4, 5 and 7.

The operational approach of updating ship time upon time zone crossings likely impacted sleep regularity for all sailors, including fixed watchstanders and dayworkers. This would be because the time change would displace non‐work hours, impacting sleep opportunities from one 24 h period to the next (see Figure 5). While this approach is not universal for underway ships and may vary by situation, the adjustment of underway ship time with time zone crossings poses a unique challenge for sailors to maintain regular sleep timing, regardless of schedule. Sailors ‘dogging’ the watch on fixed watchbills (i.e., occasionally shortening a single watch duty to impose a change of watch hours for all watchstanders on the watchbill) would likely disrupt sleep regularity, which further exemplifies the challenge of protecting sailor sleep hygiene in ways that are compatible with watchstanding practices.

There are several limitations to the current study. First, we assessed sleep regularity over a relatively short weeklong timespan. Sailors typically adhere to these watchbills for much longer timespans, over which their sleep patterns likely become more dynamic as recovery needs fluctuate (e.g., changes in sleep structure). Longer assessment of these watchbills across a range of operational settings is required to determine whether the SRIs reported here are representative of sleep regularity on these watchbills. Additionally, large sample sizes are required for the SRI to confidently detect differences between groups (Fischer et al. 2021) and variability across group sizes may have impacted our findings. Other metrics may also be suitable for the assessment of sleep regularity in this population. For instance, our data collection period was likely too short to reliably assess sleep regularity using interdaily stability (IS)—which, like SRI, does not assume consolidated sleep structure (Fischer et al. 2021). Future studies over longer timespans should investigate IS as a tool for evaluating sleep regularity in watchstanders. Finally, future research should also examine performance consequences and how they relate to changes in sleep regularity between different watchbills.

## 5 Conclusion

In conclusion, sailors from all watchbills that we evaluated were susceptible to insufficient sleep. When operational effectiveness was likely already compromised, sleep regularity assessment was shown to be a critical supplementary tool for quantifying how these watchbills differed in terms of their protection of another important dimension of sleep. Sleep regularity should continue to be assessed for watchbills in the future and should be a standardised component of relevant defence research technical reporting. Including sleep regularity assessment in studies of broader shiftwork settings may offer valuable insight into how shiftworkers are impacted by various shiftwork schedules. The findings should be considered in the context of other meaningful sleep outcomes (e.g., sleep duration), and ideally in conjunction with performance data, when evaluating schedules. Differences in sleep regularity between watchbills may be particularly valuable when the schedules tend to support a similar sleep duration. Furthermore, sleep regularity assessment may have utility in the evaluation of the effectiveness of sailor fatigue mitigation interventions (e.g., personal light treatment devices, sleep hygiene training).

## Author Contributions

**Jacob R. Guzzetti:** writing – original draft, visualization, conceptualization, writing – review and editing, methodology, formal analysis, investigation. **Panagiotis Matsangas:** data curation, conceptualization, writing – review and editing, resources, methodology, formal analysis, supervision, investigation. **Siobhan Banks:** conceptualization, writing – review and editing, methodology, supervision, investigation. **Nita L. Shattuck:** conceptualization, writing – review and editing, resources, supervision, methodology, funding acquisition, investigation.

## Ethics Statement

The Institutional Review Board of the Naval Postgraduate School approved the study.

## Conflicts of Interest

The authors declare no conflicts of interest.

## Data Availability

The data that support the findings of this study are available from the corresponding author upon reasonable request.
